# Circ_0035292 knockdown alleviates lipopolysaccharide (LPS)‐induced WI‐38 cell apoptosis and inflammatory injury

**DOI:** 10.1002/iid3.905

**Published:** 2023-06-14

**Authors:** Ying Guo, Zhouzhen Li, Chen Cheng

**Affiliations:** ^1^ Department of Pediatrics Wuhan Asia General Hospital Wuhan City Hubei China

**Keywords:** circ_0035292, infantile pneumonia, miR‐370‐3p, NF‐κB, TBL1XR1

## Abstract

**Background:**

Circular RNAs have emerged as important regulators in the pathogenesis of human diseases, including infantile pneumonia (IP). In this study, we aimed to explore the effects of circ_0035292 on lipopolysaccharide (LPS)‐treated Wistsar Institute (WI)‐38 cells.

**Methods:**

Quantitative real‐time polymerase chain reaction and western blot were executed to detect the levels of circ_0035292, microRNA‐370‐3p (miR‐370‐3p) and transducin β‐like 1X related protein 1 (TBL1XR1). Cell counting kit‐8, 5‐ethynyl‐2′‐deoxyuridine, and flow cytometry assessed cell proliferation and apoptosis. Concentrations of inflammatory factors were examined with enzyme linked immunosorbent assay kits. Dual‐luciferase reporter assay and RNA immunoprecipitation were adopted to analyze binding between miR‐370‐3p and circ_0035292 or TBL1XR1.

**Results:**

Circ_0035292 level was increased in IP patients and LPS‐triggered WI‐38 cells. Circ_0035292 knockdown rescued LPS‐mediated WI‐38 cell proliferation suppression and WI‐38 cell apoptosis and inflammation promotion. Circ_0035292 interacted with miR‐370‐3p and miR‐370‐3p directly targeted TBL1XR1. Moreover, miR‐370‐3p overexpression alleviated LPS‐induced WI‐38 cell apoptosis and inflammatory injury, which was abrogated via TBL1XR1 upregulation. Circ_0035292 absence inhibited the NF‐κB pathway.

**Conclusion:**

Knockdown of circ_0035292 rescued LPS‐triggered WI‐38 cell injury via miR‐370‐3p/TBL1XR1 axis and NF‐κB pathway.

## Graphical abstract

circ_0035292 aggravated lipopolysaccharide‐induced apoptosis and inflammatory injury in WI‐38 cells by regulating microRNA‐370‐3p/transducin β‐like 1X related protein 1/NF‐κB pathway. Our study provided a novel pathological mechanism in infantile pneumonia (IP) and might be useful for IP therapy.

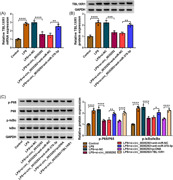

## 1 INTRODUCTION

Infantile pneumonia (IP) is one of the most common infectious diseases in infants and young children.^1^, ^2^ IP is mainly caused by bacterial or viral infection, posing a huge challenge to infants and young children's health.^1^ The current approach to IP is antiviral, antibacterial, and symptomatic supportive therapy.^3^ However, the continuous usage on antibiotics has led to an increasing resistance of pathogenic bacteria, making the disease difficult to cure.^4^ Meanwhile, the severe inflammatory reaction can damage the respiratory system of infants and young children.^5^ Therefore, further knowledge of the underlying mechanism of IP is essential to IP therapy.

During the past decades, as a large class of primarily noncoding RNA, circular RNAs (circRNAs) undergo back‐splicing events to form covalently closed‐loop structures.^6^ In mechanism, some of them can act as ceRNAs to sponge for miRNAs, thus regulating gene content.^7^ Several laboratory works discovered that dysregulated circRNAs might be implicated in the pathogenesis of pneumonia.^8^, ^9^ For example, circ‐UQCRC2 absence alleviated lipopolysaccharide (LPS)‐triggered 16HBE cell inflammation in IP via MYD88 expression.^10^ Circ_VMA21 receded LPS‐initiated Wistsar Institute (WI)‐38 cell injury via enhancing KLF4 through sponging miR‐409‐3p.^11^ Moreover, circ_0035292 (circTMOD3) interference was able to relieve LPS‐caused cell injury via miR‐146b‐3p/CXCR1 pathway.^12^ Even so, circ_0035292‐mediated potential regulatory mechanisms in IP are largely elusive.

Canonically, miRNAs modulated gene content via targeting 3′ UTR of mRNAs.^13^, ^14^ In pneumonia, multiple miRNAs have been identified.^15^ For example, miR‐483‐3p promoted inflammation and inhibited proliferation in the pneumonia cell model and could be a biomarker for pneumonia diagnosis.^16^ MiR‐874‐3p mitigated LPS‐induced inflammatory in HPAEpiC via EGR3 and NF‐κB pathways.^17^

Herein, microRNA‐370‐3p (miR‐370‐3p) shared binding sites with circ_0035292 or transducin β‐like 1X related protein 1 (TBL1XR1). Herein, based on an IP cell model constructed by LPS, circ_0035292, miR‐370‐3p, and TBL1XR1 regulatory networks in IP process was investigated.

## 2 MATERIALS AND METHODS

### 2.1 Sample acquisition

After the Ethics Committee of Wuhan Asia General Hospital authorized the study and the participants provided written informed consents, the blood samples were provided by 23 IP subjects and 19 volunteers at this hospital. Serum samples were stored at −80°C after centrifugation at 1000*g*.

### 2.2 LPS irritation

Human fibroblast cell line WI‐38 was cultured at 37°C in Minimum Essential Medium (Procell) added with 10% FBS and 1% Penicillin/Streptomycin.

Furthermore, WI‐38 cells were exposed to 0−15 μg/mL LPS (Sigma‐Aldrich). 10 μg/mL LPS for 12 h were used in the functional experiments.

### 2.3 Quantitative real‐time polymerase chain reaction (qRT‐PCR)

Total RNA was prepared according to TRIzol (Beyotime). After that, RNAs was used for cDNAs generation experiments based on PrimeScript™ RT reagent Kit (Takara) or miRNA 1st Strand cDNA Synthesis Kit (Vazyme). qRT‐PCR was executed using AceQ Universal SYBR qPCR Master Mix (Vazyme) with primer sequences (Table 1). The expression was estimated with $2^{‐\Delta \Delta C_{t}}$ strategy and normalization to glyceraldehyde 3‐phosphate dehydrogenase (GAPDH) or U6.

**Table 1:** Primers sequences used for quantitative real‐time polymerase chain reaction.

Name		Primers for PCR (5’‐3’)
hsa_circ_0035292	Forward	CAGTTTACACAGCAGGGACCA
	Reverse	GTGGTGGACTTTGATGTCTGG
TBL1XR1	Forward	TTCCTTGTGCCTCCATTCCC
	Reverse	ATATCCGGTCACCGCCAATC
miR‐370‐3p	Forward	GTATGAGGCCTGCTGGGGTGGAA
	Reverse	CTCAACTGGTGTCGTGGAG
GAPDH	Forward	AGCTCACTGGCATGGCCTTC
	Reverse	CGCCTGCTTCACCACCTTCT
U6	Forward	CGCTTCACGAATTTGCGTGTCAT
	Reverse	GCTTCGGCAGCACATATACTAAAAT

Abbreviations: GAPDH, glyceraldehyde 3‐phosphate dehydrogenase; miR‐370‐3p, microRNA‐370‐3p; TBL1XR1, transducin β‐like 1X related protein 1.

Additionally, to analyze the stability of circ_0035292, RNase R exposure at 37°C was applied for RNAs. Circ_0035292 and GAPDH content was determined.

### 2.4 Cell transfection

Si‐circ_0035292, the mimics/inhibitor of miR‐370‐3p (miR‐370‐3p/anti‐miR‐370‐3p), TBL1XR1 overexpression vector (TBL1XR1), and negative controls (si‐NC, miR‐NC, anti‐miR‐NC, pcDNA) were provided by GenePharma. Lipofectamine 2000 (Invitrogen) was employed for transfection experiments.

### 2.5 CCK‐8

For each group, 5 × 10^3^ WI‐38 cells were inoculated and incubated for 48 h. After being different treatments, cell counting kit‐8 (CCK‐8) solution (Sigma‐Aldrich) added. After 4 h, results were examined using microplate reader.

### 2.6 EdU

The 5‐ethynyl‐2′‐deoxyuridine (EdU) reagent (RIBOBIO) assessed cell proliferation. After being cultured with EdU for 2 h, the cells were fixed with paraformaldehyde, permeabilized with 0.5% Triton‐X‐100 and stained with EdU and 4′,6‐Diamidino‐2′‐phenylindole. Pictures were captured under a fluorescence microscope and EdU‐positive cells were estimated.

### 2.7 Flow cytometry

Apoptosis of WI‐38 cells was verified according to the steps of the Annexin V‐fluorescein isothiocyanate (V‐FITC)/PI kit (Beyotime). The cells were collected, washed with PBS and repeated once and removed, the cells were resuspended by adding the kit's buffer again, followed by the addition of 5 μL Annexin V‐FITC and 5 μL PI, mixed and placed at 37°C for 15 min, and cell apoptosis was verified according to flow cytometry.

### 2.8 Western blot

Referring to RIPA buffer (Beyotime), proteins in serums and cells were prepared. After separation on 10% SDS‐PAGE gel, proteins were blotted onto PVDF membranes, which were incubated with primary antibodies and interacted with secondary antibody. The end of the experiment, ECL reagent (Beyotime) was utilized for protein bands. Abcam provided secondary antibody (ab6728) and primary antibodies BCL2‐Associated X (Bax; ab32503), B‐cell lymphoma‐2 (Bcl‐2; ab182858), TBL1XR1 (ab24550), P65 (ab16502); phospho‐P65 (p‐P65; ab76302), IκBα (ab95338), p‐IκBα (ab133462), Cleaved‐caspase‐3 (ab32042), and GAPDH (ab9485).

### 2.9 ELISA

Based on enzyme linked immunosorbent assay (ELISA) kits (ab178013; ab214025; ab181421), interleukin (IL)‐6, IL‐1β and tumor necrosis factor‐α (TNF‐α) concentrations were examined.

### 2.10 Dual‐luciferase reporter

Fragments of circ_0035292 and TBL1XR1 containing binding sequences of miR‐370‐3p were introduced into pmirGLO (Promega) to construct the WT‐circ_0035292/TBL1XR1. MUT‐circ_0035292 and MUT‐TBL1XR1 were constructed via mutating the binding sequences. miR‐370‐3p mimic/miR‐NC and generated vectors were cotransfected into WI‐38 cells. After 48 h of transfection, the culture medium was changed. Cells were collected and lysed after 24 h. After centrifugation at 3500 r/min for 5 min, the supernatant was removed and sample was monitored based on Dual Luciferase Activity Assay Kit (Promega).

### 2.11 RIP

After RNA Immunoprecipitation (RIP) buffer lysis (EMD Millipore), samples were mixed with anti‐IgG or anti‐Ago2‐conjugated magnetic beads. The immunoprecipitated RNAs were determined by qRT‐PCR.

### 2.12 Statistical analysis

Software GraphPad Prism 7 analyzed data, which were presented as mean ± SD. Difference analysis was conducted via Student's *t*‐test or one‐way analysis of variance (ANOVA). Linear relationships were analyzed by Spearman's correlation coefficient analysis. It was considered significant if *p* < .05.

## 3 RESULTS

### 3.1 LPS treatment hindered WI‐38 cell proliferation and promoted apoptosis and inflammation

**Figure 1:**
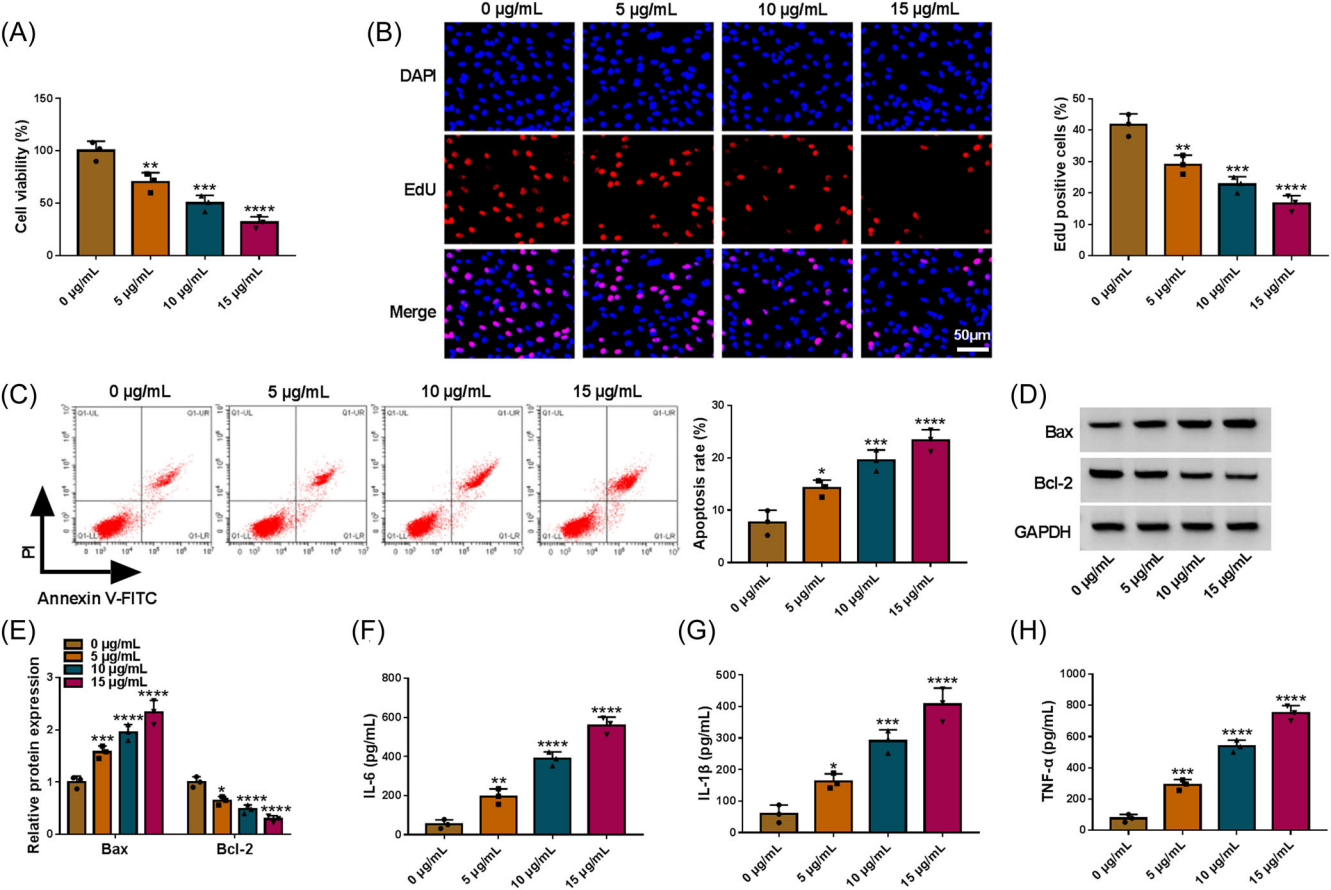
LPS induced WI‐38 cell proliferation and inflammation and repressed proliferation. WI‐38 cells were treated with different doses of LPS (0, 5, 10 and 15 μg/mL). (A and B) The proliferation of WI‐38 cells was assessed by CCK‐8 assay and EdU assay (one‐way ANOVA). (C) The apoptosis of WI‐38 cells was analyzed by flow cytometry analysis (one‐way ANOVA). (D and E) The protein levels of Bax and Bcl‐2 in WI‐38 cells were measured by western blot (two‐way ANOVA). (F−H) The concentrations of IL‐6, IL‐1β and TNF‐α in WI‐38 cells were examined with ELISA kits (one‐way ANOVA). *p < .05, **p < .01, ***p < .001, ****p < .0001. ANOVA, analysis of variance; LPS, lipopolysaccharide; TBL1XR1, transducin β‐like 1X related protein 1.

First of all, WI‐38 cells were treated with different doses of LPS (0, 5, 10, and 15 μg/mL) for 12 h. As a result, LPS diminished WI‐38 cell viability in a dose‐dependent manner (Figure 1A). Simultaneously, applying LPS dampened WI‐38 cell proliferation (Figure 1B). On the contrary, WI‐38 cell apoptosis was facilitated via LPS exposure (Figure 1C). Moreover, LPS exposure increased Bax level and reduced Bcl‐2 in WI‐38 cells in a dose‐dependent manner (Figure 1D,E). As displayed in Figure 1F−H, LPS treatment increased IL‐6, IL‐1β and TNF‐α concentrations. To sum up, LPS treatment caused fibroblast cell injury.

### 3.2 Circ_0035292 knockdown promoted LPS‐stimulated WI‐38 damage

**Figure 2:**
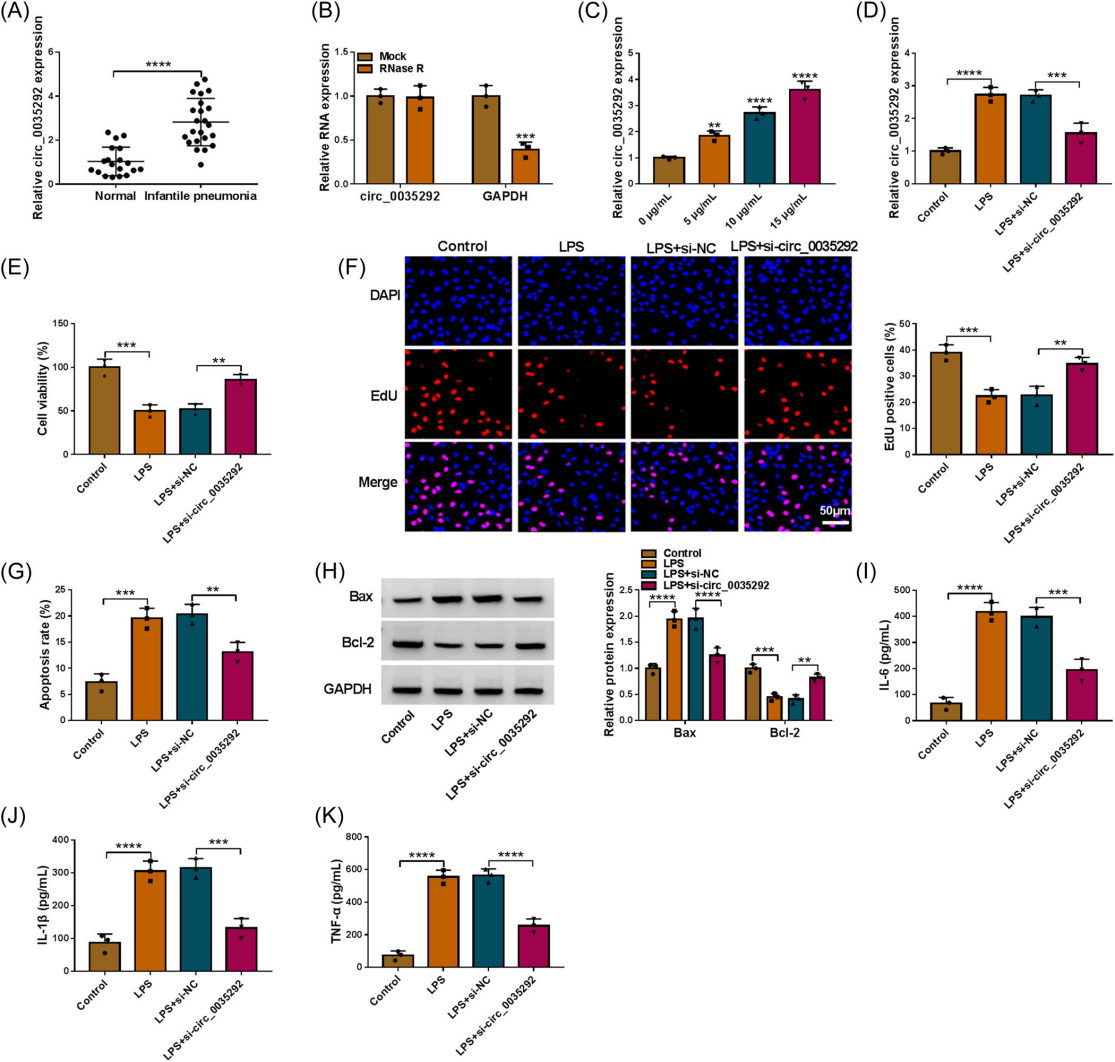
Silencing of circ_0035292 promoted cell proliferation and repressed apoptosis and inflammation in LPS‐stimulated WI‐38 cells. (A) The expression of circ_0035292 in the serums of IP patients (*N* = 23) and normal controls (*N* = 19) was determined by qRT‐PCR (Unpaired *t* test). (B) The expression of circ_0035292 and GAPDH in WI‐38 cells treated with or without RNase R was determined by qRT‐PCR (two‐way ANOVA). (C) The expression of circ_0035292 in different doses of LPS treated WI‐38 cells was detected by qRT‐PCR (one‐way ANOVA). (D−K) WI‐38 cells were assigned into 4 groups: control, LPS, LPS+si‐NC and LPS+si‐circ_0035292. (D) The expression of circ_0035292 in WI‐38 cells was determined by qRT‐PCR (one‐way ANOVA). (E and F) The proliferation of WI‐38 cells was evaluated by CCK‐8 assay and EdU assay (one‐way ANOVA). (G) The apoptosis of WI‐38 cells was analyzed by flow cytometry analysis (one‐way ANOVA). (H) The protein levels of Bax and Bcl‐2 in WI‐38 cells were measured via western blot (two‐way ANOVA). (I−K) The concentrations of IL‐6, IL‐1β and TNF‐α in WI‐38 cells were examined with ELISA kits (one‐way ANOVA). **p < .01, ***p < .001, ****p < .0001. ANOVA, analysis of variance; LPS, lipopolysaccharide; qRT‐PCR, quantitative real‐time polymerase chain reaction.

As shown in Figure 2A, circ_0035292 level was upregulated in serums of IP patients. Consistent with other typical circRNAs, circ_0035292 was resistant to RNase R digestion (Figure 2B). Circ_0035292 level was elevated in LPS‐treated WI‐38 cells in a dose‐dependent manner (Figure 2C). Moreover, applying LPS‐triggered circ_0035292 elevation in WI‐38 cells was rescued after si‐circ_0035292 transfection (Figure 2D). Repressive role of LPS treatment in WI‐38 cell proliferation was reversed by circ_0035292 silencing (Figure 2E,F). Besides, circ_0035292 absence inhibited LPS‐induced WI‐38 cell apoptosis (Figure 2G). LPS stimulation increased Bax and Cleaved‐caspase‐3 levels and decreased Bcl‐2 level in WI‐38 cells, while circ_0035292 knockdown restored the effect (Figure 2H and Supporting Information: Figure S1). In addition, LPS‐induced IL‐6, IL‐1β and TNF‐α elevation was ameliorated by circ_0035292 knockdown in WI‐38 cells (Figure 2I−K). In addition, circ_0035292 level was positively correlated with these inflammatory factors in IP subjects (Supporting Information: Figure S2). Taken together, circ_0035292 inhibited LPS‐caused WI‐38 cell injury.

### 3.3 Circ_0035292 targeted miR‐370‐3p

**Figure 3:**
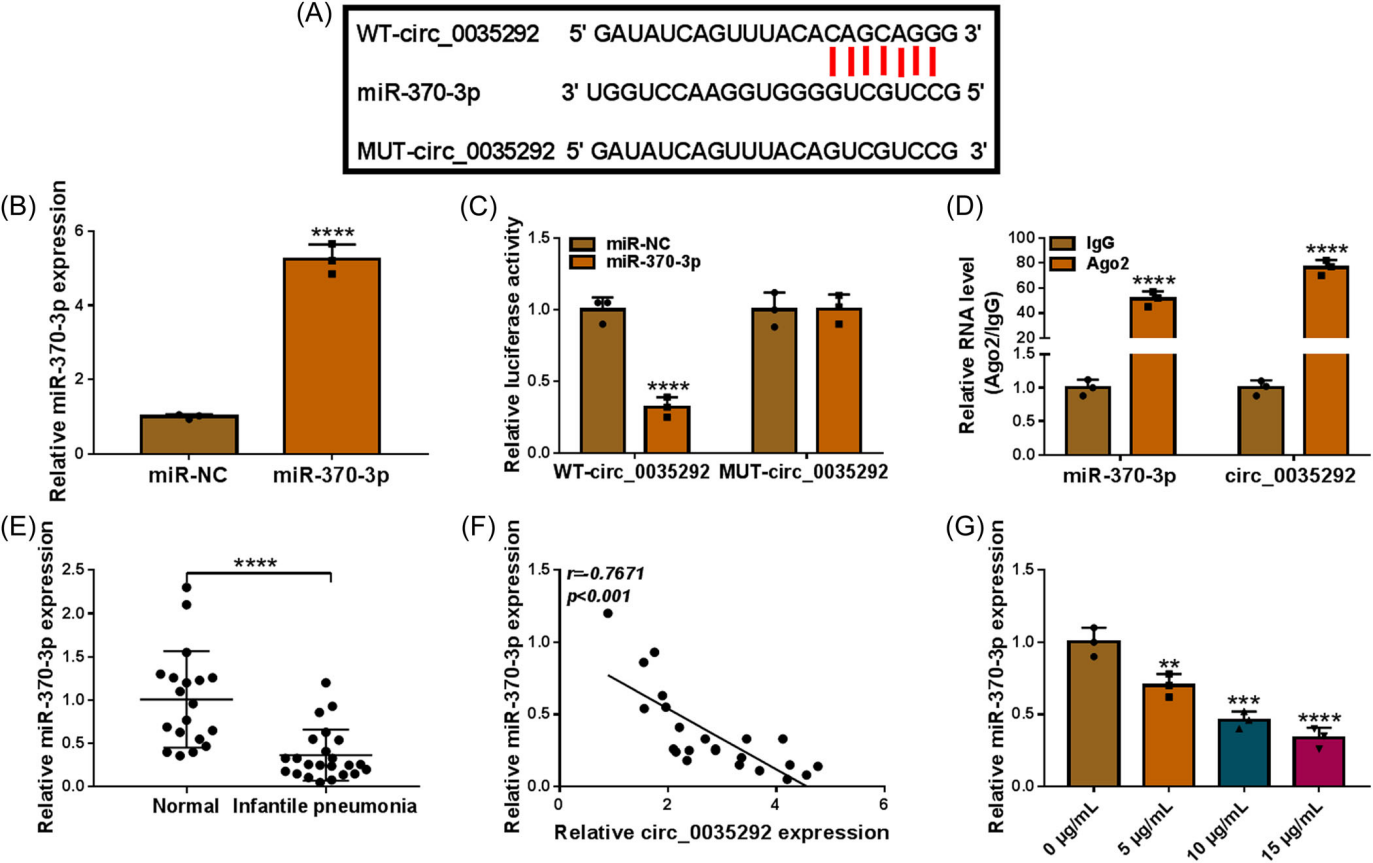
Circ_0035292 directly interacted with miR‐370‐3p. (A) The binding sites between circ_0035292 and miR‐370‐3p. (B) The expression of miR‐370‐3p in WI‐38 cells transfected with miR‐370‐3p and miR‐NC was determined by qRT‐PCR (Unpaired *t* test). (C and D) The interaction between miR‐370‐3p and circ_0035292 was demonstrated by dual‐luciferase reporter assay and RIP assay (two‐way ANOVA). (E) The expression of miR‐370‐3p in IP patients (*N* = 23) and healthy controls (*N* = 19) was detected by qRT‐PCR (Unpaired *t* test). (F) The linear correlation between the levels of miR‐370‐3p and circ_0035292 in IP patients (*N* = 23) was analyzed. (G) The expression of miR‐370‐3p in LPS‐treated WI‐38 cells was examined by qRT‐PCR (one‐way ANOVA). **p < .01, ***p < .001, ****p < .0001. ANOVA, analysis of variance; LPS, lipopolysaccharide; miR‐370‐3p, microRNA‐370‐3p; qRT‐PCR, quantitative real‐time polymerase chain reaction.

Furthermore, potential target miRNAs of circ_0035292 were analyzed by circinteractome. As presented in Figure 3A, there are continuous binding sites in the nucleotide sequences of miR‐370‐3p and circ_0035292. Overexpression efficiency was assessed and presented (Figure 3B). Overexpressing miR‐370‐3p elicited a repression in luciferase activity of WT‐circ_0035292, without affecting mutant group (Figure 3C). According to RIP results in Figure 3D, miR‐370‐3p and circ_0035292 levels were enriched in Ago2 antibody immunoprecipitated complexes versus IgG groups. miR‐370‐3p was downregulated in IP patients' serums (Figure 3E). Moreover, circ_0035292 level was inverse correlation with miR‐370‐3p in IP patients (Figure 3F). Besides, LPS treatment reduced miR‐370‐3p content (Figure 3G). Collectively, circ_0035292 interacted with miR‐370‐3p.

### 3.4 Circ_0035292/miR‐370‐3p might regulate LPS‐induced WI‐38 cell injury

**Figure 4:**
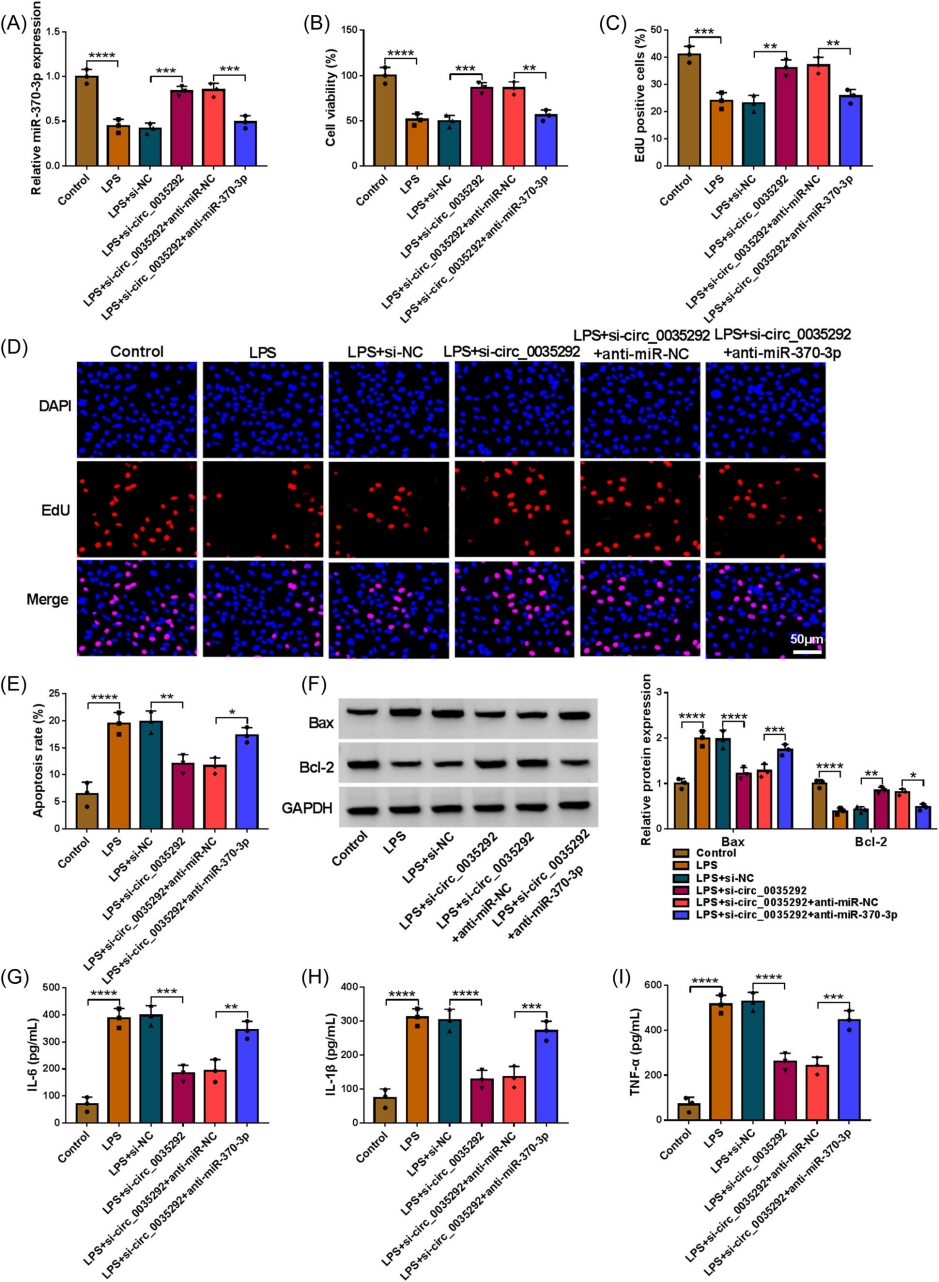
Circ_0035292 knockdown relieved LPS‐induced WI‐38 cell injury by interacting with miR‐370‐3p. WI‐38 cells were treated with control, LPS, LPS+si‐NC, LPS+si‐circ_0035292, LPS+si‐circ_0035292+anti‐miR‐NC or LPS+si‐circ_0035292+anti‐miR‐370‐3p. (A) The expression of miR‐370‐3p in WI‐38 cells was determined by qRT‐PCR (one‐way ANOVA). (B−D) The proliferation of WI‐38 cells was assessed by CCK‐8 assay and EdU assay (one‐way ANOVA). (E) The apoptosis of WI‐38 cells was analyzed by flow cytometry analysis (one‐way ANOVA). (F) The protein levels of Bax and Bcl‐2 in WI‐38 cells were measured via western blot (two‐way ANOVA). (G–I) The levels of IL‐6, IL‐1β and TNF‐α in WI‐38 cells were examined with ELISA kits (one‐way ANOVA). *p < .05, **p < .01, ***p < .001, ****p < .0001. ANOVA, analysis of variance; LPS, lipopolysaccharide; miR‐370‐3p, microRNA‐370‐3p.

Under LPS exposure, circ_0035292 knockdown markedly increased miR‐370‐3p content, and abated via miR‐370‐3p inhibitor (Figure 4A). Functionally, under LPS exposure, miR‐370‐3p inhibition partially ameliorated circ_0035292 silencing‐mediated cell proliferation enhancement (Figure 4B−D). Moreover, circ_0035292 knockdown repressed cell apoptosis, concomitant with downregulation of Bax and upregulation of Bcl‐2, which was abolished via miR‐370‐3p inhibition under LPS condition (Figure 4E,F). As examined by ELISA kits, circ_0035292 interference reduced IL‐6, IL‐1β and TNF‐α concentrations, which was partly mitigated through miR‐370‐3p downregulation (Figure 4G−I). Besides, miR‐370‐3p level was negatively correlated with these factors in IP patients (Supporting Information: Figure S2). Taken together, circ_0035292 modulated miR‐370‐3p to affect LPS‐evoked fibroblast cell injury.

### 3.5 MiR‐370‐3p directly interacted with TBL1XR1

**Figure 5:**
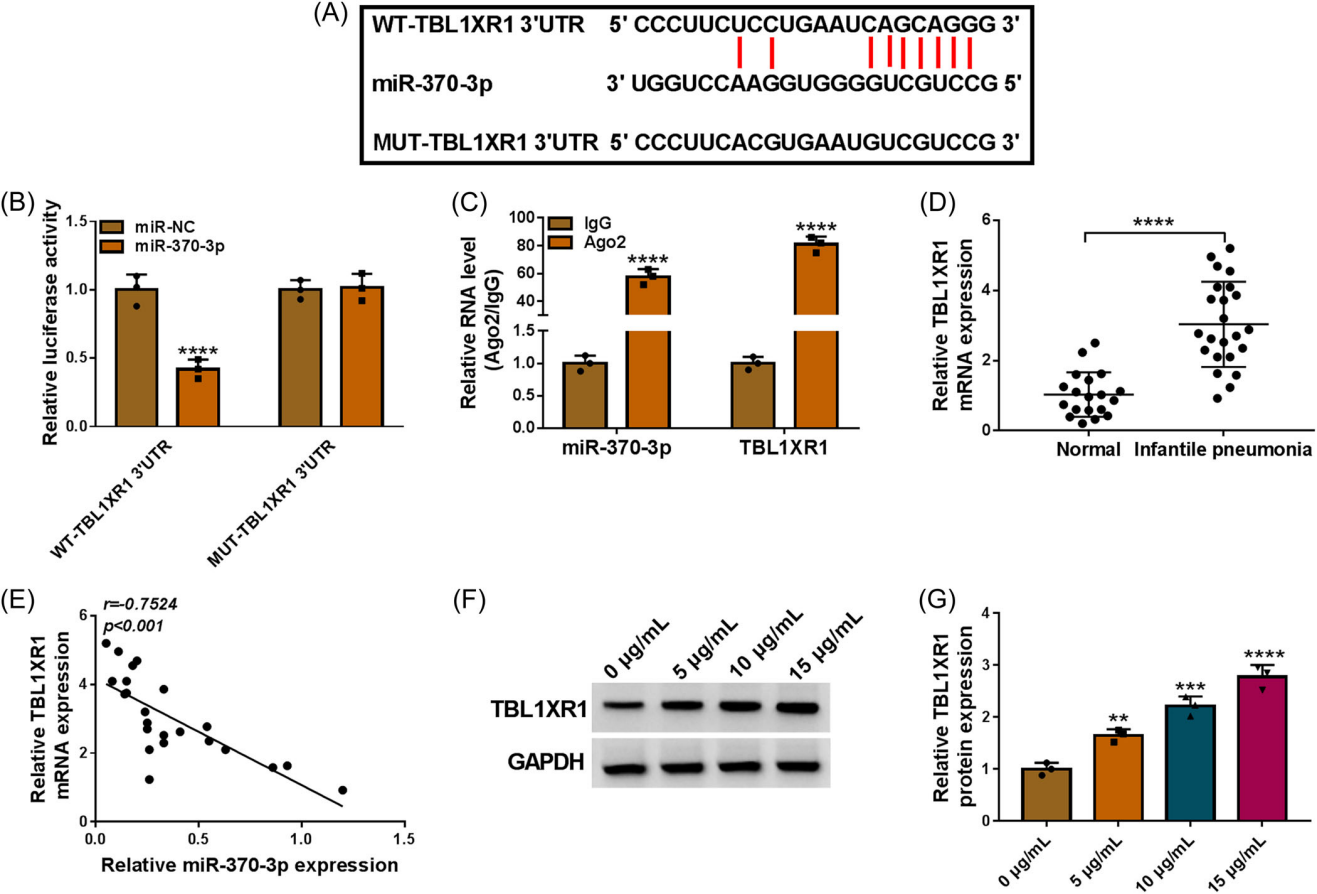
TBL1XR1 was targeted by miR‐370‐3p. (A) The binding sites between miR‐370‐3p and TBL1XR1. (B and C) The relationship between miR‐370‐3p and TBL1XR1 was verified by dual‐luciferase reporter assay and RIP assay (two‐way ANOVA). (D) The mRNA expression of TBL1XR1 in IP patients' serums (*N* = 23) and normal controls (*N* = 19) was determined by qRT‐PCR (Unpaired *t* test). (E) The linear correlation between the levels of miR‐370‐3p and TBL1XR1 in IP patients (*N* = 23) was estimated by Spearman's correlation coefficient analysis. (F and G) The protein level of TBL1XR1 in LPS‐treated WI‐38 cells was measured by western blot (one‐way ANOVA). **p < .01, ***p < .001, ****p < .0001. ANOVA, analysis of variance; LPS, lipopolysaccharide; miR‐370‐3p, microRNA‐370‐3p; TBL1XR1, transducin β‐like 1X related protein 1.

Subsequently, target genes of miR‐370‐3p were explored using starbase (https://starbase.sysu.edu.cn/starbase2/index.php). As a result, the presence of miR‐370‐3p binding sites in TBL1XR1 (Figure 5A). Enhanced miR‐370‐3p constrained the luciferase activity of WT‐TBL1XR1 3'UTR, rather than the mutant group (Figure 5B). As exhibited in RIP results, miR‐370‐3p and TBL1XR1 were increased in Ago2 RIP immunoprecipitates (Figure 5C). TBL1XR1 content was improved in IP patients' serums and negatively associated with miR‐370‐3p (Figure 5D,E). In WI‐38 cells, applying LPS markedly increased TBL1XR1 content (Figure 5F,G). Taken together, miR‐370‐3p targeted TBL1XR1.

### 3.6 TBL1XR1 might effectively overturn miR‐370‐3p‐mediated cell behaviors under LPS exposure

**Figure 6:**
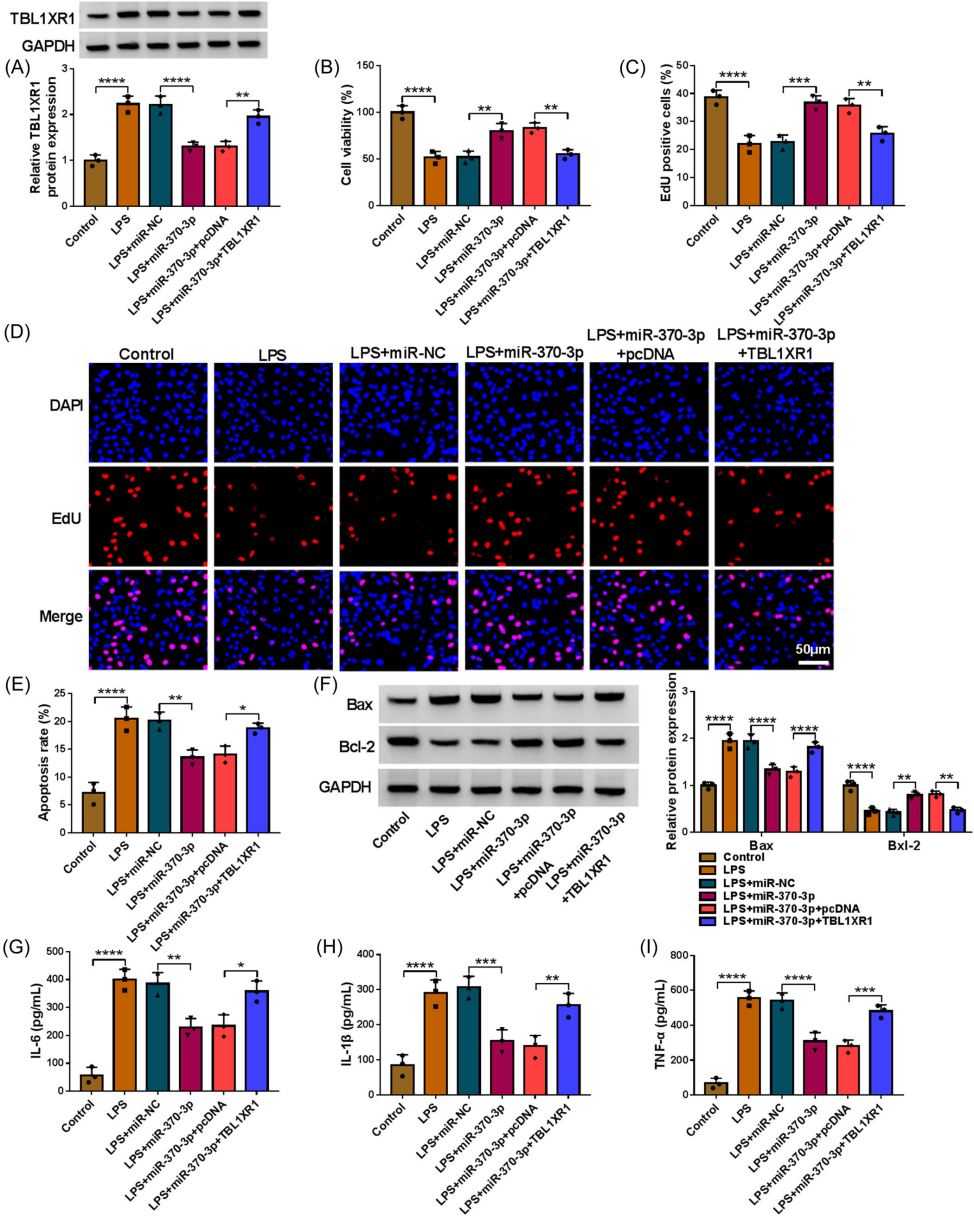
MiR‐370‐3p regulated LPS‐treated WI‐38 cell proliferation, apoptosis and inflammation by interacting with TBL1XR1. WI‐38 cells were treated with control, LPS, LPS+miR‐NC, LPS+miR‐370‐3p, LPS+miR‐370‐3p+pcDNA or LPS+miR‐370‐3p+TBL1XR1. (A) TBL1XR1 protein level in WI‐38 cells was measured by western blot (one‐way ANOVA). (B−D) The proliferation of WI‐38 cells was evaluated by CCK‐8 assay and EdU assay (one‐way ANOVA). (E) The apoptosis of WI‐38 cells was analyzed by flow cytometry analysis (one‐way ANOVA). (F) The protein levels of Bax and Bcl‐2 in WI‐38 cells were measured via western blot (two‐way ANOVA). (G–I) The concentrations of IL‐6, IL‐1β and TNF‐α in WI‐38 cells were examined with ELISA kits (one‐way ANOVA). *p < .05, **p < .01, ***p < .001, ****p < .0001. ANOVA, analysis of variance; LPS, lipopolysaccharide; miR‐370‐3p, microRNA‐370‐3p; TBL1XR1, transducin β‐like 1X related protein 1.

As displayed in Figure 6A, elevated miR‐370‐3p might dwindle TBL1XR1 protein level, which was rescued through TBL1XR1 overexpression. miR‐370‐3p elicited an apparent intension in LPS‐triggered WI‐38 cell proliferation, which was reversed via pcDNA‐TBL1XR1 (Figure 6B−D). miR‐370‐3p overexpression repressed LPS‐triggered cell apoptosis, which were ameliorated via TBL1XR1 enhancement (Figure 6E). Overexpression of miR‐370‐3p decreased Bax protein level and increased Bcl‐2 protein level in LPS‐treated WI‐38 cells, with TBL1XR1 elevation abrogated this phenomenon (Figure 6F). Under LPS condition, miR‐370‐3p might decrease IL‐6, IL‐1β and TNF‐α concentrations, but the effects were weakened via upregulating TBL1XR1 (Figure 6G−I). Additionally, TBL1XR1 level was positively associated with these factors in IP subjects (Supporting Information: Figure S2). Overall, miR‐370‐3p overexpression alleviated LPS‐evoked cell injury via targeting TBL1XR1.

### 3.7 Circ_0035292 knockdown inhibited NF‐κB pathway via miR‐370‐3p/TBL1XR1 axis

**Figure 7:**
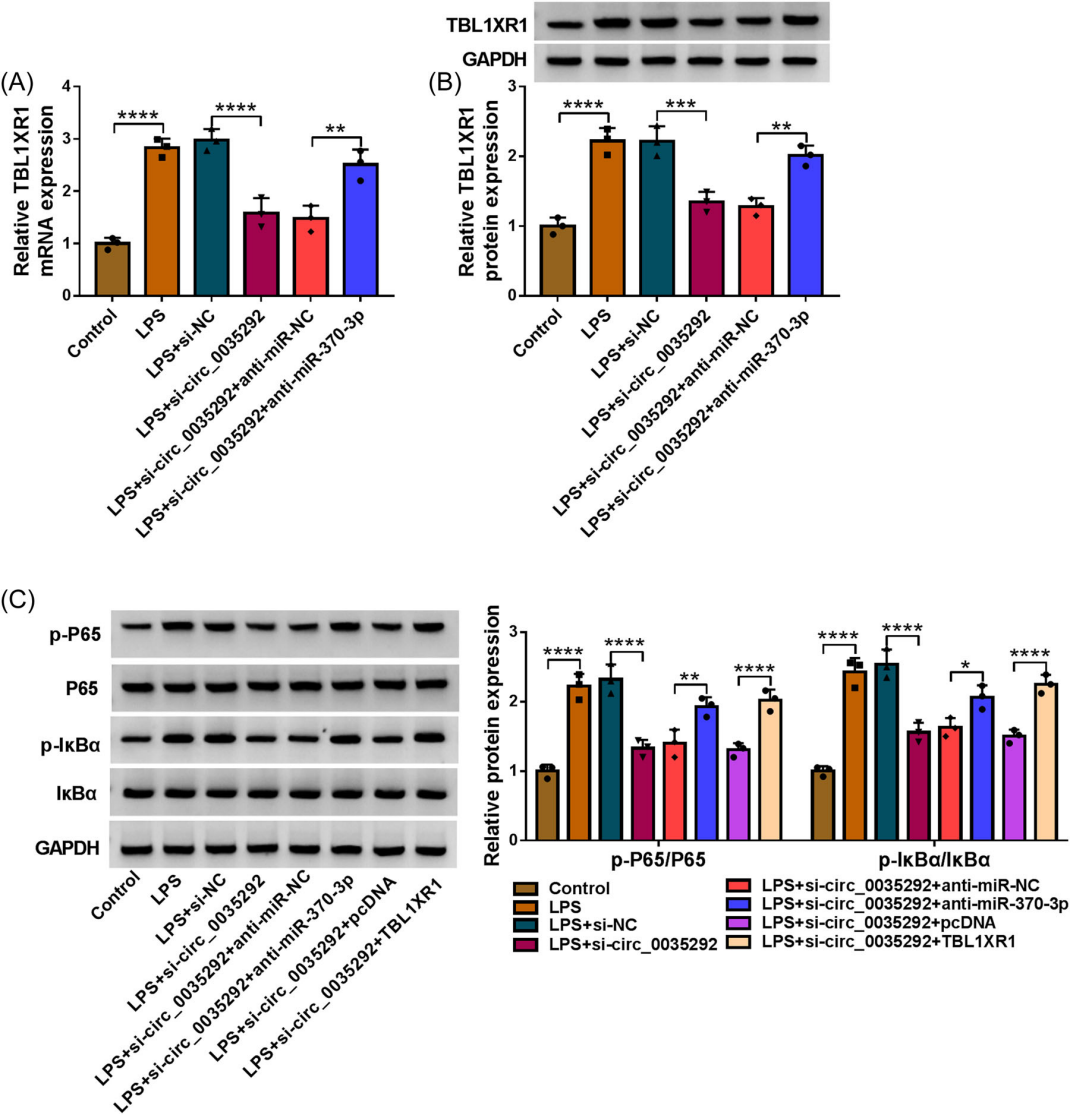
Circ_0035292/miR‐370‐3p/TBL1XR1 axis activated NF‐κB pathway. (A and B) After WI‐38 cells were treated with control, LPS, LPS+si‐NC, LPS+si‐circ_0035292, LPS+si‐circ_0035292+anti‐miR‐NC or LPS+si‐circ_0035292+anti‐miR‐370‐3p, the mRNA and protein levels of TBL1XR1 were detected by qRT‐PCR and western blot, respectively (one‐way ANOVA). (C) After WI‐38 cells ere treated with control, LPS, LPS+si‐NC, LPS+si‐circ_0035292, LPS+si‐circ_0035292+anti‐miR‐NC, LPS+si‐circ_0035292+anti‐miR‐370‐3p, LPS+si‐circ_0035292+pcDNA or LPS+si‐circ_0035292+TBL1XR1, the levels of p‐P65/P65 and p‐IκBα/IκBα were measured by western blot (two‐way ANOVA). *p < .05, **p < .01, ***p < .001, ****p < .0001. ANOVA, analysis of variance; LPS, lipopolysaccharide.

As shown in Figure 7A,B, circ_0035292 knockdown decreased TBL1XR1 content, which was restored via miR‐370‐3p absence. Besides, circ_0035292 silencing reduced the levels of NF‐κB pathway‐related proteins (p‐P65/P65 and p‐IκBα/IκBα), which were rescued via miR‐370‐3p inhibition or TBL1XR1 overexpression (Figure 7C). In summary, circ_0035292 silencing might repress NF‐κB pathway via miR‐370‐3p/TBL1XR1 axis.

## 4 DISCUSSION

The high incidence of pneumonia represents a significant challenge to public health worldwide.^18^ Inducing an inflammatory response, LPS has been extensively utilized to build IP model in vitro.^19^, ^20^ CircRNAs are vital regulators in pneumonia progression.^8^ Up to date, only a few circRNAs in pneumonia have been discovered. In the present research, the functions of circ_0035292 in IP were investigated using LPS‐evoked WI‐38 cells.

Numerous circRNAs play vital regulatory roles in pneumonia process. For instance, circ_0038467 interference restored LPS‐induced apoptosis and inflammatory damage of MRC‐5 cells through regulating miR‐195‐5p/TLR4 pathway and inactivating NF‐κB pathway.^21^ Circ_0038427 deficiency undermined LPS‐aroused inflammatory damage via decoying miR‐338‐3p.^22^ LPS‐triggered WI‐38 cell inflammation and apoptosis might be at least partially alleviated via circ_0035292 absence.^12^ Herein, circ_0035292 was abnormally increased in IP subjects. Its deficiency might overturn LPS‐elicited WI‐38 cell proliferation repression and apoptosis and inflammatory promotion.

Plentiful circRNAs can alter gene expression via adsorbing miRNAs.^7^ In this paper, circ_0035292 directly bound to miR‐370‐3p. A number of earlier studies have described that miR‐370‐3p alleviated the inflammatory damage in diverse diseases, such as sepsis‐associated acute kidney injury,^23^ acute myocardial infarction^24^ as well as pneumonia.^25^, ^26^, ^27^ Herein, miR‐370‐3p might mitigate LPS‐evoked WI‐38 cell injury. Moreover, circ_0035292 absence might receded LPS‐triggered cell damage through miR‐370‐3p. Besides, TBL1XR1 interacted with miR‐370‐3p. A previous study showed that TBL1XR1 could be targeted by miR‐103a‐3p to promote inflammatory injury in LPS‐stimulated BEAS‐2B cells.^28^ Herein, TBL1XR1 attenuated miR‐370‐3p‐mediated suppression on WI‐38 cell apoptosis and inflammation damage.

Interestingly, there have several literature exhibiting that NF‐κB pathway is associated with inflammatory response in pneumonia.^21^, ^29^ Accordingly, influence of circ_0035292 on NF‐κB pathway was explored. Our results exhibited that circ_0035292 knockdown inhibited LPS‐induced activation of NF‐κB pathway through the interaction with miR‐370‐3p/TBL1XR1. However, the present project was limited in vitro studies, and we will use a murine model to explore the novel mechanism in IP in the further.

In conclusion, circ_0035292 aggravated LPS‐triggered WI‐38 cell damage through miR‐370‐3p/TBL1XR1/NF‐κB pathway. Our study provided a novel pathological mechanism in IP and might be useful for IP therapy.

## AUTHOR CONTRIBUTIONS

Ying Guo, Zhouzhen Li, and Chen Cheng designed the concepts. Ying Guo and Zhouzhen Li performed the experiments. Ying Guo and Zhouzhen Li acquired and analyzed the data. Ying Guo and Zhouzhen Li prepared and edited the manuscript. Chen Cheng supervised the study and reviewed the manuscript. All authors approved the final edition.

## CONFLICT OF INTEREST STATEMENT

The authors declare no conflict of interest.

## Supporting information


**Figure S1. Cleaved‐caspase‐3 protein level was detected using western blot assay in WI‐38 cells treated with control, LPS, LPS+si‐NC and LPS+si‐circ_0035292** (one‐way ANOVA). **P < 0.01, ***P < 0.001.

**Figure S2. Spearman's correlation coefficient analysis was applied to evaluate the expression association**. (A‐C) Expression correlation between circ_0035292 and IL‐6, IL‐1β, or TNF‐α in IP patients was analyzed by Spearman's correlation coefficient analysis. (D‐F) Expression association between miR‐370‐3p and IL‐6, IL‐1β, or TNF‐α in IP patients was assessed using Spearman's correlation coefficient analysis. (G‐I) Expression association between TBL1XR1 and IL‐6, IL‐1β, or TNF‐α in IP patients was detected using Spearman's correlation coefficient analysis. N = 23.
