# Supplementary figures and images for: Circ_0035292 knockdown alleviates lipopolysaccharide (LPS)‐induced WI‐38 cell apoptosis and inflammatory injury
Source: Immun Inflamm Dis. 2023 Jun 14;11(6):e905. doi: 10.1002/iid3.905 (PMC10266152; doi:10.1002/iid3.905)

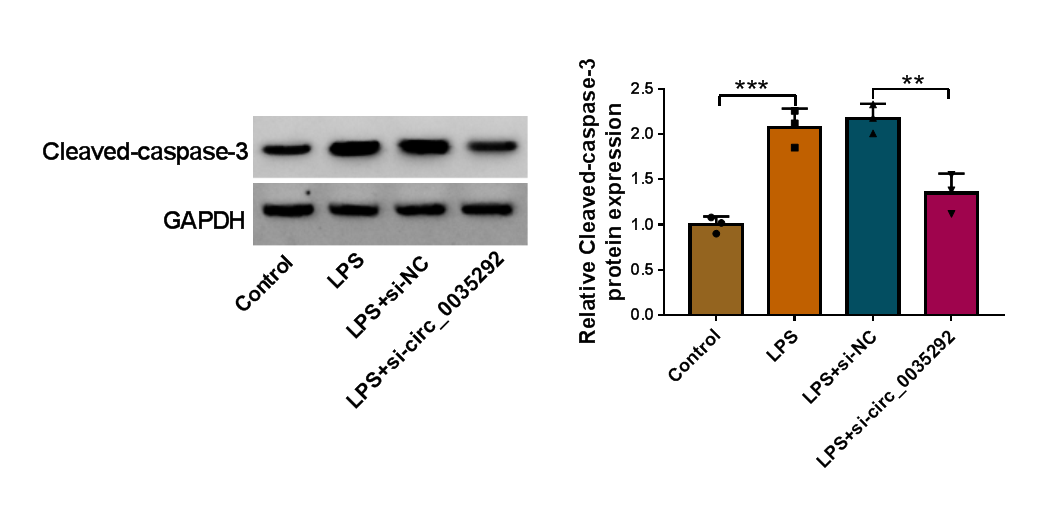

Supplement: Supplementary file 1 — Figure S1. Cleaved‐caspase‐3 protein level was detected using western blot assay in WI‐38 cells treated with control, LPS, LPS+si‐NC and LPS+si‐circ_0035292 (one‐way ANOVA). **P < 0.01, ***P < 0.001. [file IID3-11-e905-s002.tif]

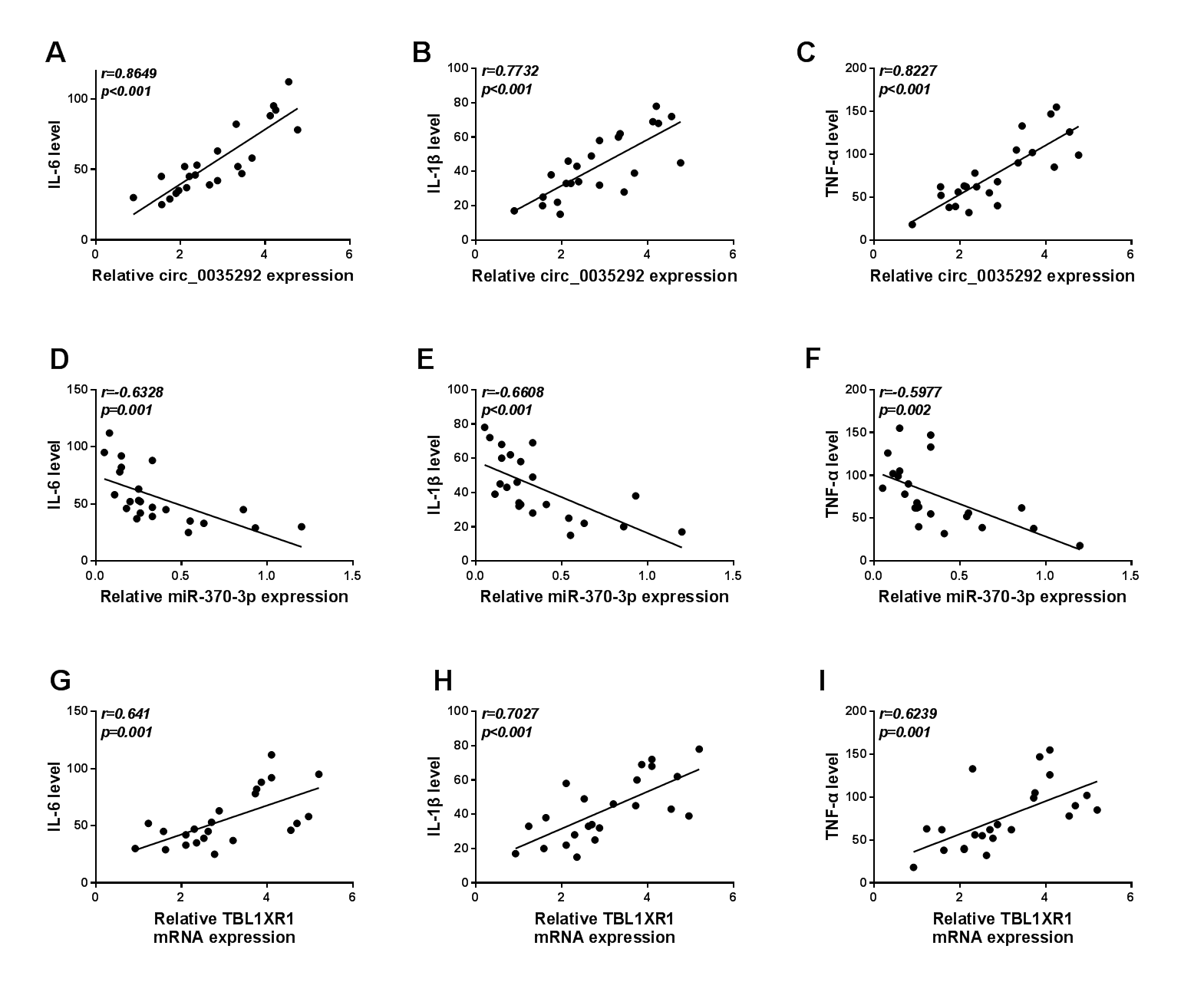

Supplement: Supplementary file 2 — Figure S2. Spearman's correlation coefficient analysis was applied to evaluate the expression association. (A‐C) Expression correlation between circ_0035292 and IL‐6, IL‐1β, or TNF‐α in IP patients was analyzed by Spearman's correlation coefficient analysis. (D‐F) Expression association between miR‐370‐3p and IL‐6, IL‐1β, or TNF‐α in IP patients was assessed using Spearman's correlation coefficient analysis. (G‐I) Expression association between TBL1XR1 and IL‐6, IL‐1β, or TNF‐α in IP patients was detected using Spearman's correlation coefficient analysis. N = 23. [file IID3-11-e905-s001.tif]
